# Representation and Learning in Neuronal Networks: A Conceptual Nervous System Approach

**DOI:** 10.5041/RMMJ.10054

**Published:** 2011-07-31

**Authors:** Danny Eytan

**Affiliations:** Network Biology Research Laboratories, Lorry Lokey Interdisciplinary Center for Life Sciences and Engineering and Pediatrics A, Rambam Medical Center, Haifa, Israel

**Keywords:** Adaptation, learning, multi-electrode array, neuronal network

## Abstract

The work presented in this review describes the use of large cortical networks developing *ex vivo*, in a culture dish, to study principles underlying synchronization, adaptation, learning, and representation in neuronal assemblies. The motivation to study neuronal networks *ex vivo* is outlined together with a short description of recent results in this field. Following a short description of the experimental system, a set of basic results will be presented that concern self-organization of activity, dynamical and functional properties of neurons and networks in response to external stimulation. This short review ends with an outline of future questions and research directions.

## MOTIVATION AND BACKGROUND

The cornerstone of the behavioral and brain science endeavors is the notion of the psychobiological transform. According to this notion, behavior is, in principle, transformable to brain states and transitions between such states on a one-to-one basis. Behavior, in this context, is thought, language, feeling, perception, learning, movement, sensing, planning, creativity, and other meanings that are attached to the word behavior in everyday use. However, the actual nature of the psychobiological transform is extremely vague. The reason for this vagueness is the apparent complexity of the biological and behavioral realms, which include an immense number of states and of transitions between states that might be relevant. Operationally, behavioral and brain scientists find themselves in a situation where it is not at all clear what biological level of organization a psychological phenomenon should be transformed to, without losing the explanatory power of the transformation.

For some researchers the state of single synapses, potentiated or depressed, with all the molecular intricacies involved, is behaviorally relevant. There are neuroscientists for whom neuronal excitability, determined by the molecular states of membrane-embedded ionic channels, is behaviorally relevant. For others, the mere fact that a cortical neuron is a target for many thousands of other input neurons, none of which is capable on its own of firing the target neuron, suggests that the states of single synapses or single neurons cannot possibly be behaviorally relevant. For these scientists, the behaviorally relevant state concept may be realized in the form of a temporally structured firing pattern, or as an average firing rate in a population of neurons. Neural network theoreticians might reason in terms of abstract attractor states, while neurophysiologists might stress, for instance, the state of a rewarding system as defined in terms of electrical or neuro-pharmacological activity. Thus, the question of what biological level of organization a psychological phenomenon should be transformed to, without losing the explanatory power of that transformation, is open.

The assumption underlying the studies reviewed here is that the most relevant level of description is the level of neuronal populations and interactions between such populations. There is a significant body of data suggesting that even the simplest kind of mammalian behavior imaginable involves at least thousands of neurons, thousands of spikes, and hundreds of thousands of synapses. Indeed, much attention has been devoted to population level descriptions, resulting in theories such as neuronal assemblies, neuronal groups, synfire chains, population codes, etc., in attempts to understand development and functionality of neural systems. As will be shown below, the study of the dynamical properties at the neuronal networks level gives hope that the gap between levels of description may be partially bridged.

The central hypothesis of neuroscience is that behavior can and should be understood in terms of representational processes in the brain. In that context, for those of us who believe that the behaviorally relevant level is that of population, Hebb’s assertion about the neuronal assembly is very appealing.^1^ Hebb pointed at the tight connection between synchronization at the population level, representation, and learning. He suggested that the “… the simplest instance of a representative process (image or idea)” is a neuronal assembly, a group of “association-area cells” that share similar static and dynamic response properties when activated through specific receptors. Moreover, viewed from a perspective of purely mathematical principles derived from the machine learning and artificial intelligence realms, any agent that can learn complex tasks must develop some kind of internal representation of the outside world in which it resides.

These and related conjectures from the fields of psychology, engineering, and neurophysiology lead to the conclusion that the function of the nervous system, at the population or neuronal network level, can be studied in terms of three axes: representation, development, and learning. Representation denotes the study of how outside objects and sensations are “encoded” by neuronal activity and how these activities interact to form higher-level complex functionality. Learning consists of the modification of these representations, their schemes, and the internal relations between them. The environment–development problem reduces to the following (rather vague) question: How does the richness of the environment experienced by a neural network during development affect its mature structure, topology, and functional capacities?

In what follows we describe the use of multi-site interaction with large cortical networks developing *ex vivo*, in a culture dish, to study basic biophysical aspects of synchronization, adaptation, learning, and representation in neuronal assemblies. We will briefly describe the experimental system, basic results regarding the self-organization of activity in this system, and the dynamical properties of neurons and networks in response to external stimulation. We show that the individual neurons and networks display very complex, history-dependent response patterns that pose constraints on possible representation schemes. Moreover, we will show the feasibility of such representation schemes and implications of their usage. Finally we will pose some future questions and research directions.

## THE EXPERIMENTAL SYSTEM: THE NEURONAL NETWORK OR ASSEMBLY

Much of the research work aimed at the fundamental issues mentioned above, at the population level, has been carried out at the theoretical level. These theories are based on physiological data from small numbers of entities (neurons, synapses) and complemented by large-scale computer simulations. Most notable of these are physical theories of artificial neuronal networks. These theories literally transformed the field of neuroscience, prompting physiologists and psychologists to think in terms of distributed functions and maps within the context of large populations of interacting elements. The ability of network theories to predict functional output from ensembles of individual noisy and unreliable elements is (most often) demonstrated numerically or (rarely) derived analytically under severely limiting simplifications. However, the simplified network units used in these theoretical studies are remote from the richness of biological neuronal entities, the complexity of their networks, and their interactions with the environment. Thus most neuronal network concepts are yet to be tested experimentally in physiological settings. This state-of-the-art points towards an acute need for controlled multi-level experimental access to large networks of real neurons over the wide range of relevant time and length scales (milliseconds to weeks; micrometers to millimeters). An ideal experimental system should serve both as a source for fresh insights as well as a natural test bed for verification or modification of existing theories. What are the requirements from an experimental network system? The system should allow for simultaneous stimulation and recordings from many individual neurons and individual synapses; long-term monitoring and manipulation of both activity and structure over the wide range of relevant time and length scales; enforcement of developmental constraints at both the structural and functional levels; access to chemical modulation; and controllability of connection between elements as well as between ensembles. Such omni-potentiality is practically impossible at the level of behaving organisms or in preparations where preformed structures are examined *in vitro* (e.g. brain slices). Here we review the use of multi-site interaction with large cortical networks developing *ex vivo*, in a culture dish, to study basic biophysical aspects of synchronization, adaptation, learning, and representation in neuronal assemblies.

Out of various alternatives, large, random, cultured networks of cortical neurons developing *ex vivo* are most appropriate experimental model systems for studying the general questions of learning and memory at the population level. An extensive survey of the properties of large, random, cortical networks developing *ex vivo* may be found in recent reviews.^2^,^3^ These networks are relatively free of predefined constraints and intervening variables, yet the electrophysiological, biochemical, and pharmacological properties of their neurons are by and large identical to neurons *in vivo*.^4^–^9^ The proportions of different cell types are practically identical to those found *in vivo*.^10^–^12^ Unlike slice preparations, the *ex vivo* developing networks are not cut out of a larger system to which their structures are particularly fitted, and in the absence of which they might function aberrantly. Indeed, alternative models, such as acute cortical slices and cultured slices, allow one to explore “what is there”, but not “how it got to be there”. The latter question is tightly related to development, and slices have a limited capacity to develop. The *ex vivo* developing model system enables extensive, multi-site sampling and manipulating of the relevant variable, that is, electrical activity.^3^,^13^–^16^ While many observables can be measured in a neural system, electrical activity is most relevant to the organization and function of networks. The *ex vivo* developing cortical network system enables non-invasive measurement procedures that interfere little with the action of universal factors. More-over, it allows for study over a wide range of time-scales (milliseconds to months).^4^,^17^,^18^

## SPONTANEOUS AND EVOKED ACTIVITY IN NETWORKS OF CORTICAL NEURONS

Our model system consists of large, random, cortical networks developing *ex vivo*. In each network there are several thousands of neurons, both excitatory and inhibitory, receiving synaptic inputs from hundreds of presynaptic cells and, in turn, affecting other neurons via heavily arborized axonal trees. The neurons are initially derived from dissociated newborn rat cortices and are plated upon a multi-electrode array (MEA) in which some 60 recording and stimulation electrodes are embedded. After plating, within hours the neurons begin to extend processes and, over a period of several weeks, the neurons form an intricate network of connections. Prior studies^3^ from other labs as well as our own showed that these networks undergo several phases of development within the first month after plating: from sporadic uncorrelated spiking activity across the network, to strongly correlated bursts, to mature partly correlated rich activity. During the same period, neurons evolve from immature cells that exhibit vigorous axonal and dendritic growth, to maturing neurons that form and break numerous synaptic connections, and, ultimately, to neurons with relatively stable and consolidated morphology. These stages and corresponding time-frames are surprisingly similar to those observed in developmental studies *in vivo*. Finally, in an extensive set of experiments, Corner and colleagues^2^ showed that the aforementioned intrinsic spontaneous activity has a critical-period time-dependent impact on the structure of neurons and their plasticity.

**Figure 1:**
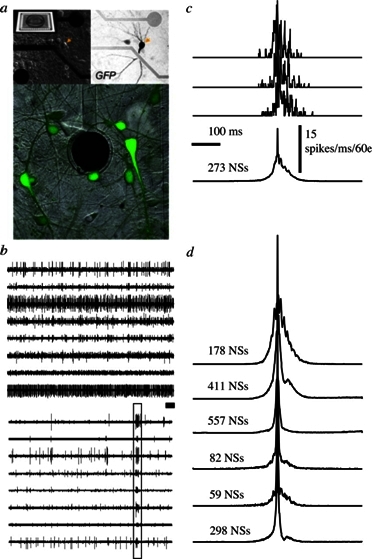
The MEA and the network spike (NS). A: Cortical network on substrate-embedded multi-electrode array. The dark circle is a 30-μm-diameter electrode. Neurons are tagged using green fluorescent protein. B: Example of spontaneous activity simultaneously recorded from eight different channels. Top: at 500 s. Bottom: higher temporal resolution of 30 s from the top panel (extracted section is depicted by a dark bar). A box marks a single event of synchronous activity. C: Top three traces show examples of individual synchronous events in terms of number of spikes recorded in 60 electrodes (1 ms time bins). The average of 273 such events (NSs) is shown. D: Example of average NSs recorded over 1 h from different networks (normalized amplitudes). Figure taken by permission from reference 21.

At later stages of functional network maturation, the global activity is characterized by complex aperiodic, synchronized bursting activity with minute-to-minute fluctuations in the probability of firing. Between these bursts, or network spikes, some tonic activity of a subset of the neurons can be also observed (Figure 1). As stated above, these stages are reminiscent of the developmental stages described from *in-vivo* recordings. Moreover, the network spike – a burst of action potentials comprising the entire networks, lasting in the order of 100 milliseconds – is remarkably similar to synchronizations recorded *in vivo* from cortices of mammals during stimulus presentation and categorization tasks.^19^,^20^

In recent works by us and others the basic properties of the network spike were described.^21^–^24^ It is a synchronized population event governed by a threshold, which follows the logistics of neuronal recruitment in an effectively scale-free connected network. The sequence of neuronal activation within these spikes is non-random and follows a hierarchy that is probably dictated by the topology of connections. We have also shown that using prior knowledge of this recruitment pattern the appearance of a network spike can be reliably predicted and used to alter and manipulate activity within and between neuronal assemblies.^21^,^25^–^28^

**Figure 2:**
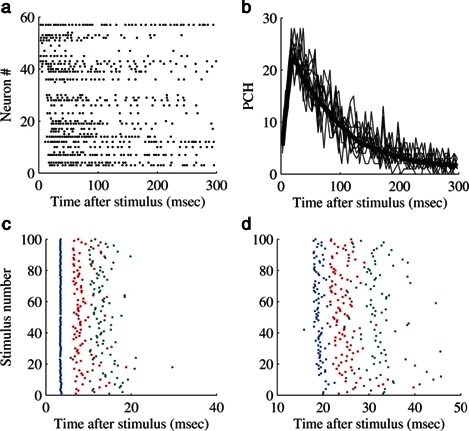
The evoked network spike (NS). All the panels are examples from a single experiment. A: An example of a single, stimulus-evoked NS. Each line is a raster plot of a single electrode. B: Population firing rate profiles of NS (population-count-histogram (PCH)). Each thin line is the histogram of a single evoked NS, binned with a 5 ms bin size; the thick black line is the average of 120 responses. All NS are evoked by the same stimulating electrode. C, D: A raster of the first three spikes of two example neurons. Here also it can be seen that while the immediate first spike is very precise, later spikes suffer from a large jitter. C: The raster is elicited from a neuron participating in the immediate response. D: The raster is created from a neuron first firing in the recruitment phase. Figure by permission from reference 38.

The effects of stimulation on these networks have also been extensively studied. It has been shown that extracellular electrical stimulation from spatially different sources elicits prototypical responses in the form of network spikes. These spikes exhibit two distinct phases of response – an early, directly activated response in which action potential latencies are well preserved and a later, “downstream” phase elicited by reverberation of activity which is very variable.^29^,^30^ Each neuron typically fires many action potentials in each network spike as it is being activated by many different propagation pathways (Figure 2).

**Figure 3:**
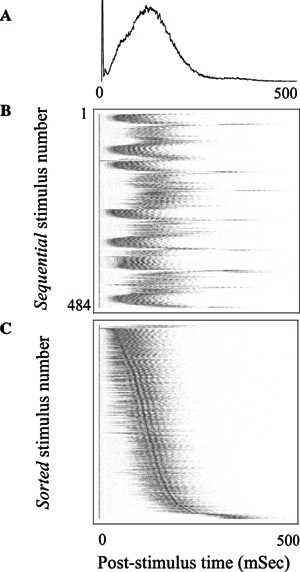
Latencies to population responses. A: Population post-stimulus time histogram (pPSTH). A total of 52 electrodes in which spikes were detected in >15% of the stimuli were considered for this analysis. The number of spikes recorded in a time window of 500 ms following each of 484 stimuli was registered in 1 ms time bins, averaged, normalized to peak, and plotted in black line; the absolute value at the peak ∼100 ms is ∼4 spikes/ms per 52 electrodes. The stimuli were applied from a single stimulation site at a frequency of 0.3 Hz. B: Horizontal lines, coded by a gray-scale in which maximal spike counts are depicted black, show the responses to each of the 484 individual stimuli. Note trial-to-trial variations. C: The individual responses of panel 3B, sorted based on their time to peak. Note the range and multiplicity of time-scales involved. Figure by permission from reference 39.

The ability to stimulate electrically at different spatial locations, different repetition rates and stimulus amplitudes, over extended periods (up to weeks) allows a detailed characterization of the input-output properties of these networks – as models for a generic neuronal assembly. Thus, we consider these networks as single entities, pooling together all the activity of the neurons comprising the network. In general there seems to be a monotonically albeit threshold-governed relationship between the stimulation amplitude and response amplitude and an inverse relation to the response latency. Moreover, it seems that as the stimulation frequency is increased, adaption processes kick in: when the stimulation frequency is “high” enough (i.e. 0.2–1 Hz) the network response initially undergoes a period of habituation, which is stimulus site-specific,^31^ but over time a complex non-trivial pattern of responsiveness emerges with response latency fluctuations exhibiting long-term correlations (Figures 2 and 3). These fluctuations are caused by processes at the synaptic levels – namely synaptic depression and facilitation,^31^ and by similar processes at the neuronal excitability level itself – as has been recently shown.^32^ Moreover, recently it has been shown that the excitation–inhibition balance strongly modulates the magnitude of these trial-by-trial variations (N. Haroush, personal communication, 2011). Thus, it seems that there is no “elementary” input-output function of these networks – rather they exhibit unstable patterns with step transitions between modes and long-term correlations in the firing statistics.

## MAPPING THE CONCEPT OF LEARNING TO THE NETWORK PREPARATION

Once the aim is to study neural mechanisms of learning, it is important to be clear about what exactly one means by “learning”. Learning can be loosely defined as a process of changing behavior in order to achieve a growing success in any a-priori task within a fixed environment. With this definition in mind, we map the concept of learning to the network preparation: The behavior, we assume, may be represented by temporal structures described in terms of associations between neuronal activities. The network is required to modulate associations between neuronal activities such that it noticeably increases the efficiency with which an input stimulus is processed and a desirable spatiotemporal firing pattern is reached.

The learning process can be artificially divided into two overlapping phases – one of exploration, that is a search in the space of possible input–output relations, and a second phase of recognition or consolidation once the “appropriate” response pattern has been reached. In the past years, there have been many publications regarding different protocols to induce plasticity in these networks^33^,^34^ (and references therein). All of these methods are based on the hypothesis that certain patterns of activation by stimulation can induce lasting changes in the network’s functional connectivity or activation pathways. What these studies mainly show is that such changes can indeed be achieved, but there are no simple “plasticity rules” at the network level, such as those discovered for single synapse in the sense of long-term potentiation (LTP), long-term depression (LTD), or spike-timing-dependent plasticity (STDP). By using measures such as conditional firing probability (CFP)^33^ or association pairs,^35^ the changes in the functional connectivity between thousands of neuronal pairs can be quantified and monitored over time. It seems that stimulation drives changes in connectivity, but the direction and amplitude of change is not easily predicted and varies between different protocols and laboratories.^30^,^34^,^36^ It does seem, however, that the “harder” the stimulation drive, the larger the change.

Using these observations, Shahaf and Marom a decade ago developed a protocol for achieving learning in these networks. By using closed loop experiments, in which these biological networks interact with a computer-controlled environment, they demonstrated a simple procedure for learning arbitrarily chosen tasks, defined in terms of neuronal firing patterns. They focally stimulated the network at a low frequency (0.3–1 Hz) until a desired predefined response was observed after a stimulus, at which point the stimulus was stopped for several minutes. Repeated cycles of this procedure ultimately led to the desired response being directly elicited by the stimulus. This was the first time that learning (not only plasticity) was demonstrated in networks or “real biological” neurons, outside the body. Since then, these results were replicated by several groups,^34^,^37^ and some constraints on the learning and its relations to spontaneous activity were defined. It should be noted, however, that these protocols are very limited in the ability to achieve a complex learning task. So far there has been no successful report, to our knowledge, of learning an arbitrary sequence of activation comprising more than two neurons or learning of two different input-output relations in one network at the same time. The reason for this failure might lie in the specific nature of the model preparations – these networks are highly interconnected, without an anatomical division into different modules, and thus it would be extremely difficult to induce a change in a subset of activation pathways without affecting the majority of the other pathways.

An attempt to study the role of neuromodulation on the activation pathways in these networks has also been made. Neuromodulators such as dopamine might have a role as a “reward” signal, thereby stabilizing “correct” activation pathways, or as a drive for change, aiding in the exploration process. Indeed a recent study^35^ showed that dopamine seems to be more of a driver for change – a single, global application can induce a lasting change in the network’s functional connectivity array. More closed loop experiments which relate the activity of the network to the application of dopamine and other neuromodulators are needed in order to define their role in the learning process.

## REPRESENTATION OF EXTERNAL INPUTS IN NEURONAL NETWORKS

While the notion that object representation is embedded in sequences of action potentials is fairly well accepted among neuroscientists, there is less agreement concerning the actual representation schemes (i.e. neuronal activity features) that carry stimulus-relevant information at the assembly level. Attempts to address this question range from *in-vivo* measurements combined with psychophysical procedures, to abstract mathematical constructs that are realized (in most cases) in numerical simulations. The results reviewed on the biophysics of the neural assembly have profound implications for the feasibility of different representation schemes or codes. It is clear that a code that relies on the exact and specific latency of neuronal response cannot serve as a basis for representation, as the response latencies fluctuate widely under repetitive stimulation, with large trial-by-trial variability. Likewise, a code based on the exact number of spikes elicited by each neuron might be irrelevant in the face of similar fluctuations in response amplitude.

**Figure 4:**
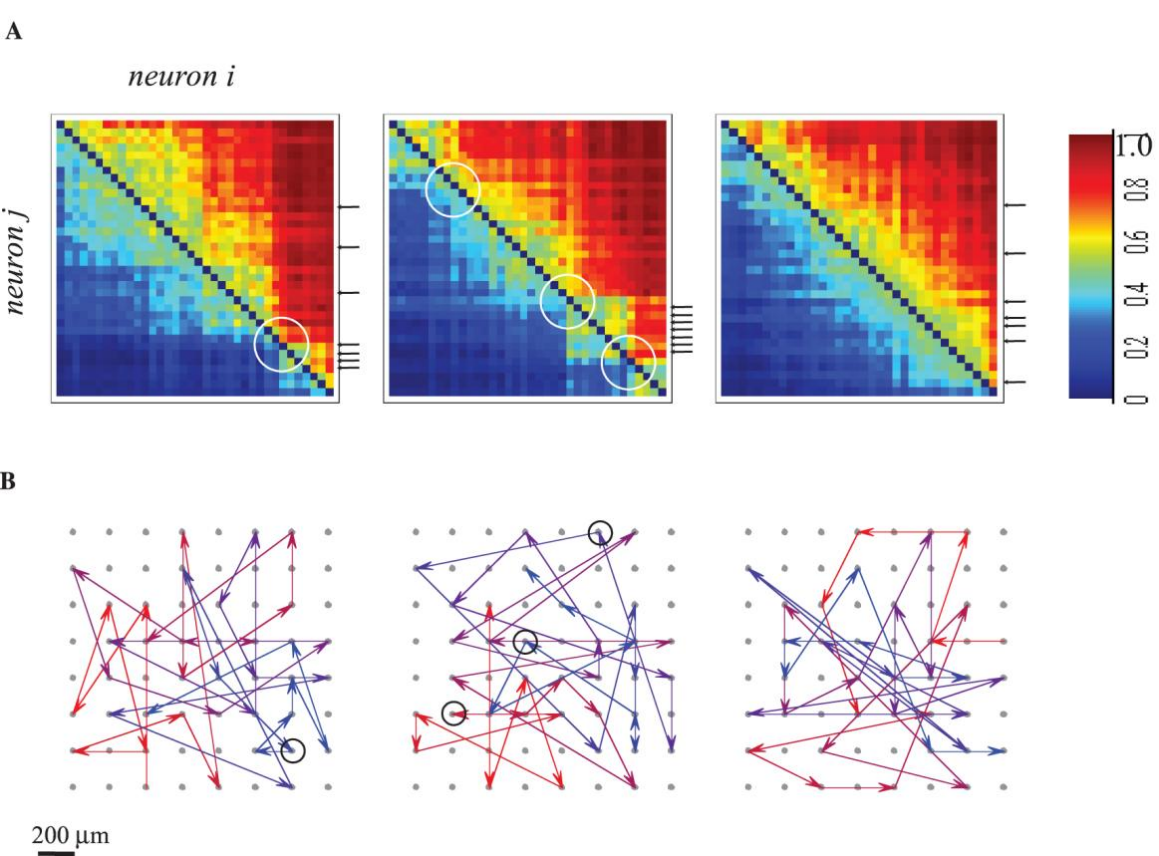
Demonstration of neuronal stations through which activity is required to pass in order to propagate further into the network. A: Pair-order probability matrices, generated from responses to three different stimulation sources of one network: The matrices (each for one of the three stimulation sources) depict the probability (color-coded) of each neuron to precede every other neuron. Neurons are presented in these matrices sorted by their average rank. White circles depict the impact of presumed bottle-necks outside the sampled area. Small black arrows to the right of the middle panel depict a cluster of neurons that tend to respond close to each other in terms of their recruitment order; each arrow indicates one of these neurons. The dispersion of these arrows in the other (right and left) panels indicates that the rank of any given neuron is stimulus site-specific. B: Activation pathways for the three sources shown in A above. The average rank vector of the responses to each source is projected onto a map of the physical locations of each electrode. Note that propagation lines that connect between electrodes that are horizontally or vertically aligned might mask each other and give the impression that a sequence has more than one end-point; to overcome this graphical problem, a color-coding for the rank of each arrow (red to blue) is used. Circles depict physical locations of neurons circled in 4A. Figure taken by permission from reference 39.

In two recent studies,^38^,^39^ we have examined the range of possible representation schemes and their feasibility in view of the intense fluctuation in the responses. We have initially shown that in spontaneously developing large-scale random networks of cortical neurons *in vitro* the order in which neurons are recruited following each stimulus is a naturally emerging representation primitive that is invariant to significant temporal changes in spike times. With a relatively small number of randomly sampled neurons, the information about stimulus position is fully retrievable from the recruitment order. It seems that this is due to the existence of propagation paths: chains of neuronal stations through which activity is required to pass in order to propagate further into the network, regardless of the status of membrane and synaptic dynamics, as can be seen in Figure 4.

In a second study, we followed the path of a stimulus reconstruction approach to compare systematically the representational efficacy of four types of popular schemes, two rate-based and two time-based: population-count histogram, spike-count, time-to-first-spike, and rank-order. We found that the nature of response in neural populations dictates strong correlations between different response features, which are a priori independent (e.g. rank order of first events and population time histogram are completely orthogonal features of a set of general spike trains^38^,^40^), resulting in high redundancy in response features. Thus, all representation schemes perform relatively well under all conditions, with an advantage to either scheme depending on the stimulus properties. Time-based representation schemes are also more stable over long periods of time, under changes induced by the long-term dynamics of the neural assembly. On the other hand, when classification between temporal features of a given stimulus source is sought, there is an advantage to rate-based representation schemes, which are more sensitive to adaptation processes, and hence contain information with regard to the history of stimulation. We have also found that overlap between groups of receptive sheath neurons (neurons that directly respond to the stimulus and serve as a source for the assembly excitation) is translated to similarity in response pattern and can be thought of as a form of generalization. These results can give an observer (a downstream cortical area?) the freedom to choose between different “schemes”, without losing much information.

The efficiency of a recruitment-order-based code enabled us to demonstrate^40^ its feasibility by constructing a biological toy model, a realized Braitenberg Vehicle II.^41^ This is a continuously moving Lego robot that is equipped with two ultrasonic sensors that transmit their input to a large-scale network of real, cultured biological cortical neurons. The task of the agent (the Lego apparatus together with the biological network) is to avoid running into obstacles in a static environment, and it succeeds flawlessly by using the input from its sensors to drive the network while the output to the motors is dictated by a rank-order-based code. The agent performs perfectly in the sense that it succeeds in its avoidance task. Importantly, no learning is involved; the representations of stimuli from the ultrasonic eyes are fixed by the rank-order solely which is preset into the algorithm a priori.

## OPEN QUESTIONS AND OUTLOOK

So far we have shown that a neuronal network developing *ex vivo* can serve as a model for a neuronal assembly. In the past years researchers in this field have studied the basic biophysical dynamical properties of such a system, its adaptation and representation capacity and have begun to hit the constraints of exploration and learning in such networks. However, the mammalian brain is composed of a hierarchy of assemblies, and it seems that this modular structure, combined with the properties of its constituents is what enables the complex behavior observed at the level of the organism. Another key theme, neglected so far, is the interplay between the environment and the developing nervous system – it is evident from *in-vivo* studies that initial and early life experiences greatly affect the potential for learning and function in humans and mammals in general. There are dual effects between the organism and the environment in both structural (anatomical) and functional terms.

Endeavoring to understand how complex function and behavior can arise from and be mapped to the neural substrate, we envision the next stages in constructing a model for a conceptual nervous system. This will be a system of modular networks, each as complex as the ones described above. These modules will be accessible to the researcher both in terms of recording their activity and also by the ability to control their physical and chemical environments. Being able to connect these modules both by electrical and biological (via axonal and dendritic pathways) means in arbitrary patterns, we can achieve “anatomy” and study its role in the creation of function. We will study the modes of activity generated by coupling two (or more) modules and their dependence on various parameters such as latency, strength, bandwidth, and filter properties of the connections. We will explore how a modular structure affects learning and what is the role of neuromodulation in such structures. The relations between the connection topology and the functional properties can also be studied, and much more.

Finally, we envision studying the effects of the environment during the development of such neuronal systems. We hypothesize that enriched environments will give rise to a broader range of structural and dynamical measures (such as axonal/dendritic arbors, connectivity characteristics, synaptic sizes and strengths, cellular and population excitability status). We expect that these, in turn, will lead to enhanced functional capacities. These developmental experiments, and the rest of the above plan, have yet to be completed.

## Abbreviations:

CFP: conditional firing probability;
MEA: multi-electrode array.
